# Inhibition of JCPyV infection mediated by targeted viral genome editing using CRISPR/Cas9

**DOI:** 10.1038/srep36921

**Published:** 2016-11-14

**Authors:** Yi-ying Chou, Annabel Krupp, Campbell Kaynor, Raphaël Gaudin, Minghe Ma, Ellen Cahir-McFarland, Tom Kirchhausen

**Affiliations:** 1 Department of Cell Biology, Harvard Medical School, and Program in Cellular and Molecular Medicine, Boston Children’s Hospital, Boston, MA 02115, USA; 2 Department of Neuroimmunology, Biogen, Cambridge, MA 02142, USA; 3 Department of Pediatrics, Harvard Medical School, Boston, MA 02115, USA

## Abstract

Progressive multifocal leukoencephalopathy (PML) is a debilitating disease resulting from infection of oligodendrocytes by the JC polyomavirus (JCPyV). Currently, there is no anti-viral therapeutic available against JCPyV infection. The clustered regularly interspaced short palindromic repeat (CRISPR)/CRISPR-associated protein 9 (Cas9) system (CRISPR/Cas9) is a genome editing tool capable of introducing sequence specific breaks in double stranded DNA. Here we show that the CRISPR/Cas9 system can restrict the JCPyV life cycle in cultured cells. We utilized CRISPR/Cas9 to target the noncoding control region and the late gene open reading frame of the JCPyV genome. We found significant inhibition of virus replication and viral protein expression in cells recipient of Cas9 together with JCPyV-specific single-guide RNA delivered prior to or after JCPyV infection.

The JC polyomavirus (JCPyV), a member of the *Polyomaviridae* family, is the causative agent of a rare and debilitating demyelinating disease termed progressive multifocal leukoencephalopathy (PML) caused by infection and destruction of oligodendrocytes in the central nervous system (CNS)^1^,^2^. PML can develop in the context of immunodeficiency and treatment with immunomodulatory drugs^3^. Indeed, it is one of the most common CNS-related diseases in AIDS, affecting 5% of the HIV-1 positive patients. The incidence of PML has also risen to 0.2–0.4% in patients receiving immunomodulatory therapies^4^,^5^.

JCPyV infection is common in the general population infecting approximately 50% to 80% of the population^6^. JCPyV persists asymptomatically in the kidney and is shed in urine in about one third of the infected population^7^,^8^. The route of viral dissemination from the initial site of infection to the CNS in the context of immunosuppression remains unknown^9^,^10^,^11^,^12^, but it has been proposed to be mediated by hematopoietic cells crossing the blood brain barrier^13^.

JCPyV has a nonenveloped, icosahedral capsid containing a circular double-stranded DNA genome divided into three regions; the early region encoding the regulatory T antigens (small t antigen, large T antigen and T’ proteins); the late region encoding the capsid proteins; and the non-coding control region (NCCR) which contains the viral origin of DNA replication and the transcriptional promoters of the early and late genes^14^,^15^. The regulatory T antigens play critical roles in initiation of viral DNA replication and the transcription of the late genes (Agno and capsid proteins VP1, VP2 and VP3)^16^.

The type II bacterial clustered regularly interspaced short palindromic repeat (CRISPR)/CRISPR-associated 9 (Cas9) system (CRISPR/Cas9) is a powerful tool for editing cellular genomes^17^,^18^,^19^. The Cas9 endonuclease together with a small guide RNA (gRNA) can introduce double strand breaks in targeted DNA in a sequence-specific manner^20^. Similar to the original function of CRISPR, a microbial nuclease system serving as a defense mechanism against invading phages and plasmids, CRISPR/Cas9 has now been employed to specifically target mammalian viral genomes, including those of human papillomaviruses (HPV), Hepatitis B virus (HBV), Epstein-Barr virus (EBV) and HIV-1 (Reviewed in refs 21 and 22). In these cases, the CRISPR/Cas9 system introduced double strand breaks in the viral DNA genome associated with mutational inactivation of viral genes resulting in the inhibition of viral protein production and viral DNA replication. The CRISPR/Cas9 system was used to target viral genomes in cells latently infected with viruses, such as EBV and HIV^23^,^24^,^25^ or in cells with integrated viral genomes, such as HBV, HPV or HIV provirus^26^,^27^,^28^,^29^,^30^,^31^,^32^,^33^. In these cases, the copy number of targeted viral genomes in the cells were relatively low compared to actively replicating viral DNAs^23^,^26^,^27^,^28^,^29^,^30^,^31^,^32^,^33^. When inhibition of *de novo* virus infection by the CRISRP/Cas9 system was demonstrated for HBV, virus infection was done in cells stably expressing CRISPR/Cas9 system^27^. Similarly, cells expressing Cas9 and gRNAs targeting the T antigen region of JCPyV genome were less permissive to JCPyV infection^34^. In both cases, however, CRISPR/Cas9 together with specific gRNAs were already present in the cells prior to the introduction of viral genomes; it is therefore possible that the viral genome was targeted before initiation of DNA replication thus being able to restrict viral growth with high efficiency.

Here we extended these studies and explored whether the CRISPR/Cas9 system introduced into cells after acute viral infection could also be effective to restrict virus replication. We used cells acutely infected with JCPyV and then exposed them to CRISPR/Cas9 specifically targeted to the genome of JCPyV. We identified regions in the JCPyV genome susceptible to DNA cleavage by CRISPR/Cas9 and found that when these cleavages failed to be properly repaired by the host cell, JCPyV replication was significantly reduced. This observation suggests that CRISRP/Cas9 together with gRNAs specific for the JCPyV genome can be used to inhibit JCPyV infection when introduced into cells already infected with JCPyV.

## Results

### Cells stably expressing JCPyV specific gRNAs are resistant to Mad1 JCPyV infection

**Figure 1:**
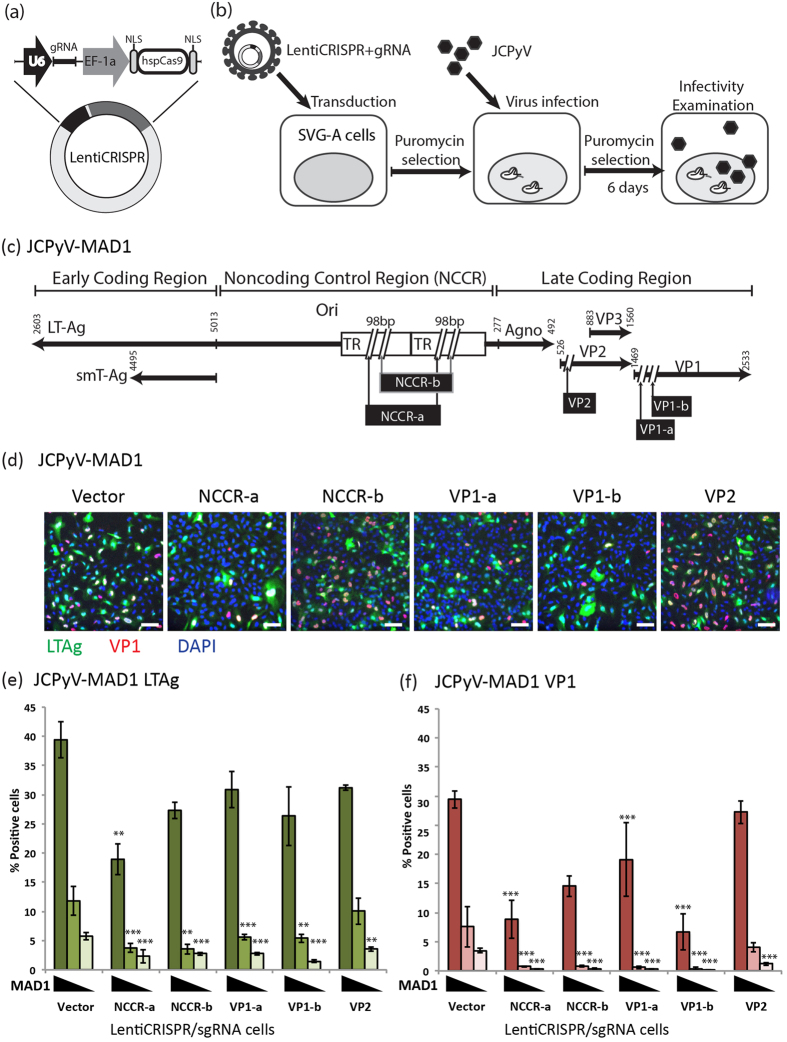
Expression of Cas9 and JCPyV specific gRNA inhibited JCPyV infection. (**a**) Depiction of the LentiCRISPR/gRNA plasmid design. EF-1a: Human elongation factor 1 alpha promoter, NLS: nuclear localization signal, hspCas9: human codon-optimized *streptococcus pyogenes* Cas9. (**b**) Experimental outline. SVG-A cells transduced with LentiCRISPR/gRNA virus with indicated gRNA were selected with puromycin and then infected with JCPyV-MAD1. (**c**) JCPyV specific gRNA target sites in JCPyV-MAD1 genome. (**d**) Immunofluorescence images showing the expression of LTAg (green) and VP1 (red) in SVG-A cells expressing Cas9 and the indicated gRNA at day 6 post-infection (highest virus inoculation dose in (**e**,**f**)). Scale bar = 100 μm (**e**) Quantification of the percentages of LTAg positive cells. (**f**) Quantification of the percentages of VP1 positive cells. Triangle represents the inoculation dose of JCPyV-MAD1 virus (5 × 10^7^ genome copies/ml, 2.5 × 10^7^ genome copies/ml, 1.25 × 10^7^ genome copies/ml). **One-way ANOVA, p value < 0.01; ***one-way ANOVA, p value < 0.001. N = 3.

To identify regions of the JCPyV genome susceptible to targeted genome editing by CRISPR/Cas9, we designed 5 different gRNAs specific to the JCPyV genome (Table 1) within the NCCR (NCCR-a and NCCR-b) and the open reading frame encoding the capsid proteins VP1 (VP1-a and VP1-b) and VP2 (Fig. 1c), respectively. The NCCR of the rearranged JCPyV strain MAD1 contains a 98 bp tandem repeat, therefore the gRNA target sequence is present twice for both NCCR targets (NCCR-a and NCCR-b) (Fig. 1c). These regions were chosen to target the origin and regulatory region for viral gene expression contained in the NCCR region and the open reading frames encoding two structural proteins essential for successful generation of virus particles. The target sequences were selected based on highly evaluated scores for predicted specificity and low off-target potential using the online CRISPR design tool (http://www.genome-engineering.org/crispr/).

We first determined whether the JCPyV genome could be successfully targeted by CRISPR/Cas9 in SVG-A cells, first transduced with LentiCRISPR/gRNA viruses (schematically represented in Fig. 1b) and then infected with JCPyV-MAD1. Virus infectivity, indirectly estimated by using immunofluorescence staining to determined the number of cells expressing the viral proteins large T antigen (LTAg) and VP1, was used to assess if Cas9 and the JCPyV-specific gRNAs already present in the cells could target incoming JCPyV genomes and reduce virus infection (schematically shown in Fig. 1c). As expected, the number of cells positive for LTAg was dependent on the dose of input JCPyV-MAD1 in control cells stably expressing CRISPR/Cas9 without gRNA (Fig. 1e, vector: 39 ±3%, 12 ± 3% and 5% ±1% of cells). LTAg expression in cells exposed to the lowest two doses of JCPyV-MAD1 was susceptible to the inhibitory effect of all LentiCRISPR/gRNAs tested. When we increased the amount of virus inoculated, we observed a significant decrease in the number of cells expressing LTAg only in cells transduced with LentiCRISPR/gRNA-NCCR-a (representative images in Fig. 1d and quantitative data in Fig. 1e), suggesting that gRNA-NCCR-a was most potent amongst the other gRNAs tested (Fig. 1e). A similar analysis carried out to follow the effect of LentiCRISPR/gRNAs on VP1 instead of LTAg expression also showed that targeting the NCCR, VP1 and VP2 regions hindered late viral protein production, with gRNA-NCCR-a and gRNA-VP1-b being the most potent (Fig. 1f). From both set of results, we concluded that the extent of the inhibitory effects of the LentiCRISPR/gRNAs inversely correlated with the amount of JCPyV used for infection.

**Figure 2:**
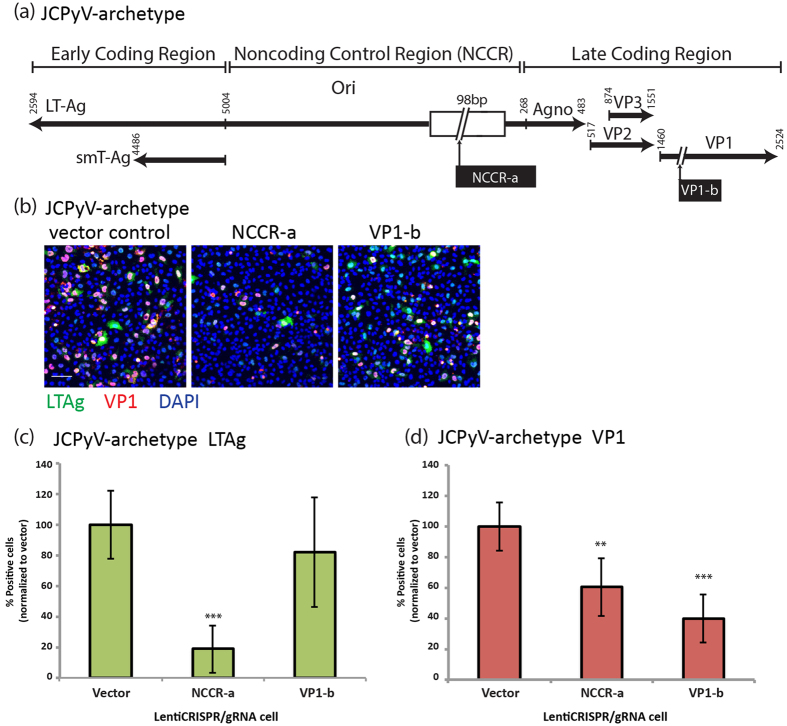
Expression of Cas9 and JCPyV specific gRNA inhibit JCPyV-archetype virus infection. (**a**) Positions of JCPyV specific gRNA target sites in the JCPyV -archetype genome. (**b**) SVG-A cells puromycin selected for Cas9 and gRNA expressions were infected with JCPyV -archetype virus (7.5 × 10^8^ genome copies/ml) for 6 days. Immunofluorescence images showing LTAg (green), VP1 (red) positive cells are presented. Cell nuclei were stained with DAPI (blue). Scale bar = 100 μm (**c**) Quantification of the percentages of LTAg and (**d**) VP1 positive cells are shown. **One-way ANOVA, p value < 0.01. ***One-way ANOVA, p value < 0.001. N = 2.

A similar decrease in the number of cells infected with JCPyV-archetype containing a non-rearranged NCCR (Fig. 2a) was achieved by expression of LentiCRISPR/gRNA-NCCR-a or LentiCRISPR/gRNA-VP1-b (Fig. 2b–d). As with JCPyV-MAD1 infection, gRNA-NCCR-a was more potent than gRNA-VP1-b in inhibiting the expression of LTAg for JCPyV-archetype virus (Fig. 2b,c).

### Effect of JCPyV specific gRNAs on the production of JCPyV transcripts and viral proteins

**Figure 3:**
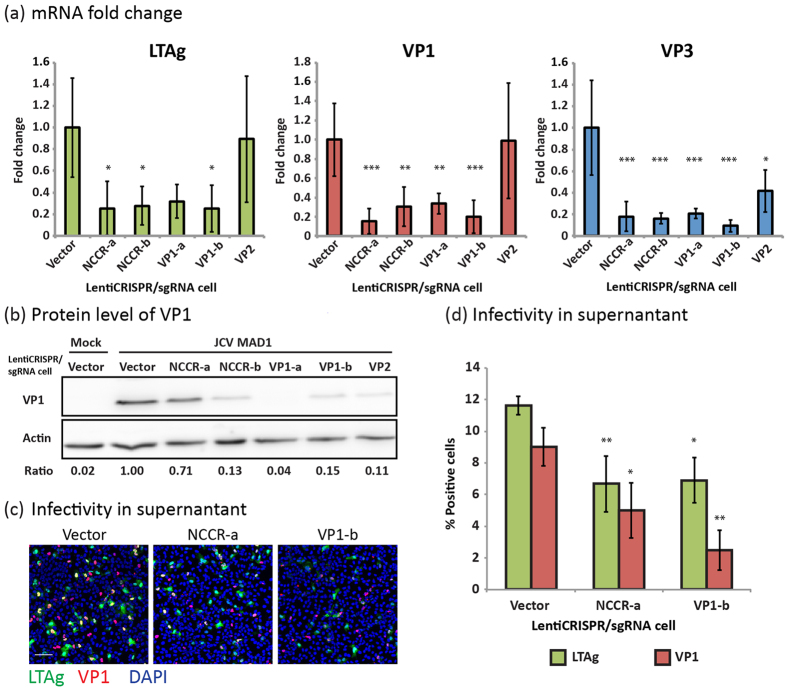
Expression of Cas9 and JCPyV specific gRNA inhibited productive JCPyV infection. (**a**) Real-time PCR analysis showing the mRNA level of LTAg, VP1 and VP3 genes in different LentiCRISPR/gRNA stably expressing cells at day 3 post-infection. Fold changes of the mRNA is normalized to the vector control. *One-way ANOVA, p value < 0.05; **one-way ANOVA, p value < 0.01; ***one-way ANOVA, p value < 0.001. N=3 (**b**) Western blot analysis showing the level of VP1 in different LentiCRISPR/gRNA stably expressing cells at day 5 post-infection. Intensity Ratio between Actin and VP1 were measured using ImageJ and presented below the blots. (**c,d**) SVG-A cells were infected with supernatant collected from JCPyV-MAD1 infected LentiCRISPR/gRNA cells for 6 days. (**c**) Immunofluorescence images showing the expression of LTAg (green) and VP1 (red) in SVG-A cells and (**d**) the Quantification of the percentages of LTAg, VP1 positive SVG-A cells are shown. **One-way ANOVA, p value < 0.01.

Except for the LentiCRISPR/gRNA targeting VP2, SVG-A cell expression of all the other LentiCRISPR/gRNAs significantly reduced the amount of mRNA of LTAg, VP1 and VP3 as determined by quantitative PCR (qPCR) (Fig. 3a). LentiCRISPR/gRNA targeting VP2 partially reduced the amount of VP3 transcript; this was the expected result given that alternative splicing from a common mRNA generates VP2 and VP3 mRNAs. VP1 protein production determined by western blot analysis was also reduced in LentiCRISPR/gRNA cells (Fig. 3b). These results agreed with the outcome of the infectivity experiments described above (Fig. 1).

### Effect of JCPyV specific gRNAs on the production of infectious viral particles

SVG-A cells were incubated with supernatant containing newly released virus particles collected from cells first transduced with either LentiCRISPR/gRNA-NCCR-a or LentiCRISPR/gRNA-VP1-b and then infected with JCPyV-MAD1. Infectivity was assayed by counting the number of LTAg and VP1 positive cells 6 days post infection. LentiCRISPR/ gRNA-NCCR-a or LentiCRISPR/gRNA-VP1-b cells generated 50% less infectious particles (Fig. 3c,d). Viruses produced from LentiCRISPR/gRNA-VP1-b cells yielded fewer VP1 positive cells as compared to those from LentiCRISPR/gRNA-NCCR-a or control cells, suggesting the existence of defective viral genomes that could express LTAg normally but not VP1. It is also possible that a fraction of JCPyV genomes in the LentiCRISPR/gRNA-VP1-b cells contained VP1 frame-shifts or deletions generated by aberrant DNA repair of Cas9/gRNA-VP1-b cleavage that nevertheless could still be amplified by replication and then packaged into the progeny virus particles.

### Cleavages of the JCPyV genome mediated by LentiCRISPR/gRNAs system resulted in incorrect genome repair

**Figure 4:**
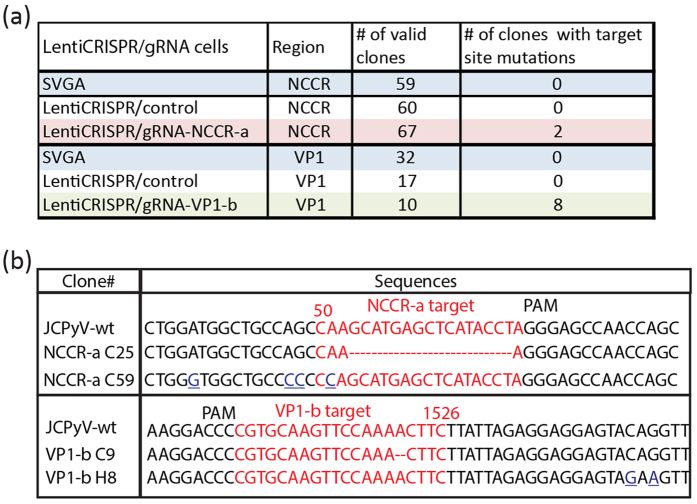
Expression of Cas9 and JCPyV specific gRNA mediated targeted cleavage in JCPyV genome. (**a**) JCPyV genome from LentiCRISPR/gRNA stably expressing cells was extracted and the regions around the indicated target were amplified by PCR reactions. PCR products were clones and 50–70 colonies from each condition were sequenced. The chart showing the number of mutant colonies identified from the sequencing analysis. (**b**) Examples of deletions or mutations identified from the sequencing analysis for JCPyV genome from LentiCRISPR/gRNA-NCCR-a and LentiCRISPR/gRNA-VP1-b cells.

We used DNA sequence analysis to confirm that inhibition of JCPyV infectivity was a direct consequence of the combined effect of the specific targeting of the Cas9 endonuclease to the JCPyV genome and its incorrect repair by the host cell. JCPyV genome was introduced into LentiCRISPR/gRNA cells by transfection of re-circularized JCPyV genome amplified in *E.coli*. At 5-day post transfection, DNA was extracted from the cells and digested with DpnI enzyme to eliminate transfected genome plasmids of bacterial origin and preserve the amplified JCPyV genome. DNA sequencing of the replicated JCPyV genome was carried out with DNA extracted from SVG-A, SVG-A-LentiCRISPR-control, SVG-A-LentiCRISPR/gRNA-NCCR-a and SVG-A-LentiCRISPR/gRNA-VP1-b cells. Since the NCCR region is vital for the replication of viral genome, the cleavage and aberrant repair of the NCCR will abrogate the synthesis and amplification of the mutant genome in the cells, resulting in a population of viral genome bias towards the intact ones. Indeed, JCPyV genome sequence reads from the SVG-A LentiCRISPR/gRNA-NCCR-a showed that 2 out of a total of 67 contained a deletion or a point mutation in the NCCR-a target region (data summarized Fig. 4a and DNA sequence shown in Fig. 4b). JCPyV genomes with mutations in the VP1 ORF are likely to be able to replicate and accumulate in the cells, making the mutations prominent to detect. As expected, 8 of 10 sequence reads contained deletions or point mutations in the VP1-b region from JCPyV genomes extracted from SVG-A-LentiCRISPR/gRNA-VP1-b cells (data summarized in Fig. 4a and examples of DNA sequence shown in Fig. 4b). These mutations were specific and required presence of the appropriate gRNAs as shown by absence of mutations in control SVG-A cells either alone or just transduced with LentiCRISPR (data summarized in Fig. 4a). Thus, Cas9 together with gRNA-NCCR-a or gRNA-VP1-b mediated targeted cleavage of the JCPyV genome that when incorrectly repaired by the host cells caused reduced viral gene expression, infectivity and virus production.

### Inhibition of established JCPyV infection by CRISPR/Cas9

**Figure 5:**
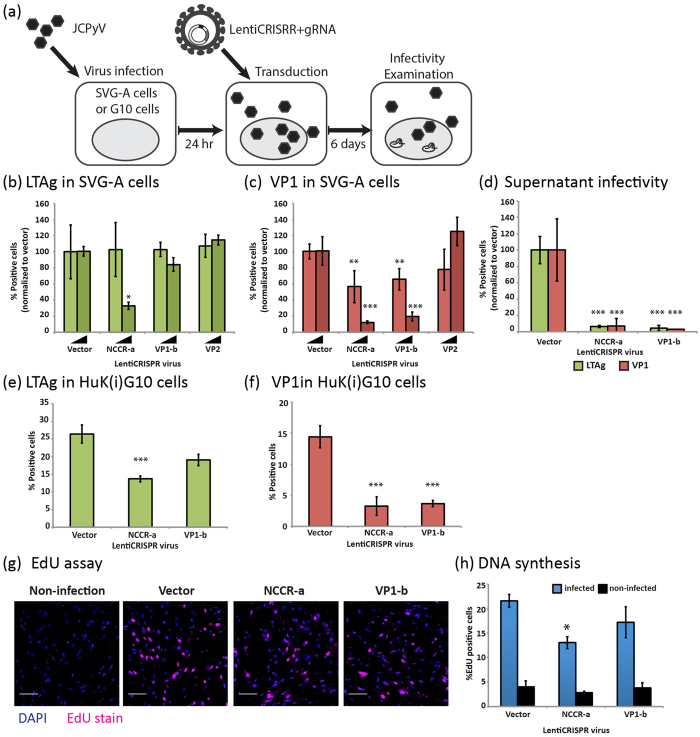
JCPyV specific CRISPR-Cas9 system inhibited established JCPyV infection. (**a**) Experimental scheme. SVG-A cells or HuK(i)G10 cells were infected with JCPyV-MAD1 for 24 hours and then were transduced with LentiCRISPR/gRNA viruses expressing different gRNAs. The level of virus infection was examined at day 6 post-infection. (**b,c**) Infected SVG-A cells were transduced with two different doses of LentiCRISPR/gRNA viruses, the percentages of (**b**) LTAg cells and (**c**) VP1 positive cells are shown. The inoculated genome copies concentration ratio of JCPyV-MAD1 and LentiCRISPR/gRNA virus were 10:1 and 40:1. The inoculation dose of JCPyV-MAD1 was 5 × 10^7^ genome copies/ml. (**d**) SVG-A cells were infected with supernatant collected from JCPyV-MAD1 infected cells transduced with LentiCRISPR/gRNA viruses (genome copy concentration ratio of JCPyV-MAD1: LentiCRISPR/gRNA = 10:1) for 6 days. The Quantification of the percentages of LTAg, VP1 positive SVGA cells are shown. ***One-way ANOVA, p value < 0.001. N = 2 (**e,f**) HuK(i)G10 cells were infected with JCPyV-MAD1 as illustrated in (**a**), the percentages of (**e**) LTAg and (**f**) VP1 positive cells are shown. ***One-way ANOVA, p value < 0.001. (**g,h**) DNA replication efficiency of the JCPyV virus genome is measured using EdU assay. (**g**) Fluorescent images of virus infected cells after Click-iT reactions are shown. Scale bar = 50 μm (**h**) Quantification of the percentages of EdU positive cells are shown for infected and non-infected cells. Non-infected HuK(i)G10 was included in the assay as a control indicating the background signal of EdU assay due to cellular DNA replication. *t-test, p value < 0.05.

We then tested whether CRISPR/Cas9 targeted to the viral genome could prevent further viral growth in cells already infected with JCPyV. As described below, we found that transduction of LentiCRISPR/gRNAs specific for the JCPyV genome in glial derived SVG-A cells or human fetal kidney derived hTERT transformed HuK(i)G10 cells already infected with JCPyV prevent viral replication and subsequent expression of LTAg and VP1. These cells were first infected for 24 hours with JCPyV-MAD1, allowing for virus replication and then transduced with LentiCRISPR/gRNAs specific for NCCR-a, VP1-b or VP2. The effect on JCPyV infectivity was assessed six days post-infection using immunofluorescence by determining the number of cells expressing LTAg or VP1 (schematic protocol depicted in Fig. 5a).

As shown above with SVG-A cells first transduced with LentiCRISPR/gRNA-NCCR-a (Fig. 1) and then infected with JCPyV-MAD1, we also found reduction in the number of SVG-A or HuK(i)G10 cells expressing LTAg when exposed to LentiCRISPR/gRNA-NCCR-a after JCPyV-MAD1 infection (Fig. 5b,e) while expression of LTAg was not influenced by LentiCRISPR/gRNA-VP1-b (Fig. 5d,f). Similarly, cells targeted with either LentiCRISPR/gRNA-NCCR-a or LentiCRISPR/gRNA-VP1-b displayed a significant reduction in the number of cells expressing VP1 (Fig. 5c,f). These inhibitory effects on LTAg, VP1 expression also strongly reduced the release of infectious virus particles (Fig. 5d). In all cases, the extent of inhibition reflected the amount of LentiCRISPR/gRNAs used for transduction.

We confirmed that LentiCRISPR/gRNA transduction of HuK(i)G10 cells already subjected to JCPyV-MAD1 infection also inhibited viral DNA synthesis, by determining the extent of EdU incorporation into newly synthesized viral DNA. As shown in Fig. 5g,h, lentiCRISPR/gRNA-NCCR-a virus transduction significantly reduced the amount of newly synthesized viral DNA when compared to cells transduced with the LentiCRISPR control virus, analyzed 7-day post JCPyV infection. In contrast, lentiCRISPR/gRNA-VP1-b transduction reduced virus DNA synthesis to a lesser extent consistent with the modest reduction in LTAg expression (Fig. 5g,h). We observed diffuse EdU incorporation in non-infected HuK(i)G10 cells indicating that the punctate EdU signal detected in JCPyV infected cells originated from viral DNA synthesis (Fig. 5g,h).

## Discussion

It has been suggested that DNA double strand breaks introduced by the CRISPR/Cas9 system leading to subsequent mutations due to unfaithful DNA repair by the host cells can be applied in the treatment of diseases, including interference with virus infections^21^,^35^. Specifically, it has recently been shown that the use of virus specific gRNAs can result in the mutational inactivation of viral genomes of viruses such as HPV, HBV, HSV, EBV and HIV-1, mostly by targeting the integrated viral genomes or viral DNA in latently infected cells^21^,^22^,^23^,^24^,^25^,^26^,^27^,^28^,^29^,^30^,^31^,^32^,^34^,^36^,^37^,^38^,^39^. Here we show that it is also possible to use the CRISPR/Cas9 system to inhibit acute virus infection. Our results with specific targeting of the JCPyV genome demonstrated that Cas9 expression alongside virus genome specific gRNAs significantly reduced viral infectivity not only prior JCPyV infection but importantly, also following JCPyV infection.

We showed that amongst the five different gRNAs selected, two gRNAs targeting the NCCR (NCCR-a) and VP1 (VP1-b) displayed strong anti-JCPyV effects. The effective gRNA-NCCR-a target sites are located within the tandem repeat region of the prototype JCPyV-MAD1 genome. Since gRNA-NCCR-a can also restrict the infection of JCPyV-archetype virus, containing one instead of two gRNA-NCCR-a target sites, it is likely that recognition and cleavage of one of the two target sites in JCPyV-MAD1 by the CRISPR/Cas9 is sufficient to mediate JCPyV inhibition. Since the NCCR region contains all the promoters and enhancer elements required for viral gene transcription, disruption of this region should affect both the early and late gene expression. Indeed, we found that gRNA-NCCR-a decreased expression of both the early gene LTAg and the late gene VP1 (Fig. 1e,f).

Presently, there is no therapeutic available to specifically treat JCPyV infection and most of the reported JCPyV inhibitors generically target the host cell such as retrograde trafficking inhibitors that can inhibit early entry steps of the virus^40^. Since JCPyV persists asymptomatically in an estimated 50–80% of the adult population^41^ and causes PML by active infection of oligodendrocytes in the CNS, it is of importance to develop strategies to diminish viral genomes already present in the host cells. Of importance, we uncovered that transduction of LentiCRISPR/gRNA viruses 24-hours post JCPyV infection significantly reduced JCPyV infectivity. Thus, the CRISPR/Cas9 system is able to inhibit an established JCPyV infection. Previously, Wollebo *et al*., showed that stable expression of Cas9 and gRNAs targeting the N-terminal region of small-T antigen is able to inhibit viral DNA replication and they also demonstrated the ability of Cas9/gRNA to edit viral DNA integrated in the genome^34^. We now show that the CRISPR/Cas9 system can also be used to inhibit on-going viral DNA replication in acutely JCPyV infected cells.

We confirmed the specificity of the inhibition by showing the ability of the CRISPR/Cas9 system to cleave incoming JCPyV genome, which is not integrated into the cellular genome (Fig. 4). The ability of the CRISPR/Cas9 system to restrict the JCPyV life cycle when delivered after virus infection has occurred, demonstrating the potential of this type of approach as a possible anti-viral strategy to fight against latent and active JCPyV infection. An efficient way of CRISPR/Cas9/gRNA delivery remains to be developed before further clinical consideration. For example, it has been proposed that adeno-associated virus (AAV) with optimized Cas9 expression vectors may be another feasible way to deliver CRISPR/Cas9/gRNA *in vitro* and *in vivo*^21^,^42^,^43^,^44^. Nevertheless, the use of the CRISPR/Cas9 system may be limited by the potential to generate JCPyV mutants of enhanced infectivity due to aberrant DNA double strand repair. Given that JCPyV strains isolated from PML patients contain genomic rearranged NCCR regions, demonstrated to increase viral gene expression and replication^45^,^46^, the application of CRISPR/Cas9 to JCPyV infection must be approached with caution.

In summary, we developed a CRISPR/Cas9 system capable of inhibiting already established JCPyV infection. We identified several gRNAs with particular two gRNAs (NCCR-a and VP1-b) able to mediate efficient anti-viral effects. We showed that pre-existence of CRISPR/Cas9 and appropriate gRNAs in cells stably transduced with LentiCRISPR/gRNA viruses rendered the cells resistant to initial JCPyV infection. Importantly, actively replicating JCPyV was also restricted upon the delivery of CRISPR/Cas9 and gRNAs with LentiCRISPR/gRNA viruses at 24-hours post JCPyV infection. Together, these data provide evidence that a JCPyV specific CRISPR/Cas9 platform might serve as a valuable research tool to study JCPyV genomics as well as a tool to restrict the JCPyV life cycle.

## Material and Methods

### Cell lines and antibodies

SVG-A cells kindly provided by Walter J. Atwood were maintained in minimum essential medium (MEM; 10–010-CV, Mediatech) supplemented with 10% fetal bovine serum (FBS), incubated at 37 °C and 5% CO_2_ in humidified incubators. 293FT cells used for lentivirus production were cultured in DMEM (10565–042, Life Technologies) supplemented with 10% FBS and 1 mg/ml G418, incubated at 37 °C and 5% CO_2_ in humidified incubators. HuK(i)G10 cell line was derived by transduction of human fetal kidney cells with a lentiviral vector expressing human telomerase reverse transcriptase (hTERT) (Applied Biological Materials #G200). HuK(i)G10 was selected from single-cell colonies isolated by limiting dilution for permissivity to JCPyV-archetype virus infection. HuK(i)G10 cells were maintained in RenaLife Complete media at 37 °C and 5% CO_2_ in humidified incubators. The antibodies used for immunofluorescence staining are: PAB2000 or PAB2003 specific for JCPyV large T antigen (gift from Richard Frisque), BIIB048 and chPAB597 (gift from Ed Harlow) specific for JCPyV VP1. Secondary antibodies: anti-mouse IgG Alexa Fluor 647 (A21236, Life technologies); Alexa Fluor 594 goat anti-human IgG (A11014, Invitrogen, Molecular Probes).

### Viruses

JCPyV-MAD1 virus (type 1Av (accession #J02226.1)) was produced by transfection of 293FT cells with pMAD1 JCPyV genome (Richard Frisque, Pennsylvania State University) circularized by ligation after EcoR1 digestion. JCPyV-archetype virus was produced using the modified pMAD1 plasmid in which the prototype NCCR sequence was replaced by the NCO1 fragment of an archetype NCCR (accession # AY121915).

### LentiCRISPR/gRNA viruses targeting JCPyV genome

Cas9 and gRNA was delivered into JCPyV permissive cells with high efficiency using a lentiviral vector (LentiCRISPR)^47^ encoding Cas9, a gRNA and a puromycin selection marker (Fig. 1a) was kindly provided by Dr. Feng Zhang.

The gRNAs target sequences in the JCPyV genome were designed using an online CRISPR design tool (http://www.genome-engineering.org/crispr/). Insertion of the gRNA targeting nucleotide sequence was performed as described^47^. LentiCRISPR viruses were produced according to protocols accessible online at http://www.bu.edu/dbin/stemcells/protocols.php with minor modifications. Briefly, 12 μg of LentiCRISPR plasmid was co-transfected with 1.2 μg of VSV-g, 0.6 μg of gag/pol, 0.6 μg of rev and 0.6 μg of tat expression plasmids into 293FT cells in 10 cm dish using 45 μl of lipofectamine 2000 (11668027, Invitrogen). Supernatant containing the LentiCRISPR virus was collected 72 hours post transfection and centrifuged at 3000 rpm, at 4 °C for 10 min to remove cell debris. The supernatant was then filtered through 0.45 µm filter (28144–007, Acrodisc Syringe Filter, VWR) and aliquots were stored at −80 °C before use.

### Virus infections and immunofluorescence analysis of JCPyV infection

SVG-A cells stably transduced with LentiCRISPR/gRNA viruses were plated in 384 well plates with 1500 cells per well 24 hours before JCPyV infection. The cells were then infected with JCPyV-MAD1 (5 × 10^7^ genome copies/ml) or JCPyV-archetype viruses (7.5 × 10^8^ genome copies/ml) in MEM supplemented with 2% FBS. The cells were fixed at the indicated times with 3.7% formaldehyde in 1× PBS for 10 minutes. Cells were then washed and permeablized with 0.1% TritonX-100 in 1× PBS for 5 minutes followed by a brief wash with 1× PBS, then blocked with 1× PBS supplemented with 5% bovine serum albumin (BSA) for 30 minutes and stained with BIIB048 or chPAB597 and PAB2003 for 1 hour at room temperature. Cells were washed 3 times with 5% BSA in 1× PBS and then incubated with goat anti-human Alexa-Fluor 594 (A11014, Invitrogen, Molecular Probes) and rabbit anti-mouse Alex-Fluor 647, Samples were washed 3 times with 5% BSA in 1× PBS and stained with DAPI for 2 minutes before imaging. The number of infected cells were counted in samples imaged using a spinning disc confocal microscope^48^. Images were acquired at 10x magnification and the analysis was performed using ImageJ. Binary masks were created for the DAPI, LTAg and the VP1 channels, respectively. The masks from the LTAg and DAPI channels were then logically multiplied, resulting in new binary image representing LTAg positive nuclei. A similar procedure was performed to obtain binary images to identify VP1 positive nuclei. The Analyze Particle plugin module of ImageJ was then used to determine the total numbers of nuclei, LTAg positive nuclei, and VP1 positive nuclei in a total of 25 imaged fields per well for each sample.

### Detection of JCPyV gene expression by Quantitative PCR

SVG-A cells stably expressing LentiCRISPR/gRNAs were infected with JCPyV-MAD1 (5 × 10^7^ genome copies/ml) for 3 days. The viral mRNA was extracted using RNeasy Plus Mini Kit (Qiagen). Real-time PCR analyses were performed using the TaqMan system with Express One-step Superscript qRT-PCR-kit (Invitrogen), following the manufacturer’s protocol. In brief, each 20 μl reaction contained 40 ng of RNA template; 10 μl of qPCR SuperMix Universal; 0.25 μl of 10 μM forward and reverse primers; 0.25 μl of 5 μM TaqMan probe and 0.4 μl ROX reference dye. Thermal cycling began with a step of 30 min at 48 °C followed by 95 °C for 10 min and 40 cycles of 95 °C 15 sec and 60 °C for 1 min, using Applied Biosystems StepOnePlus real-time PCR system. Three biological replicates were done for each condition and the level of GAPDH was analyzed for normalization. The sequences of the primer used were: (1) VP1 forward: 5′ GGGACATGCTTCCTTGTTACAGT 3′; (2) VP1 reverse: 5′ ATTTCCACAGGTTAGATCCTCATTTAG 3′; (3) VP1 FAM probe: 5′ TGGCCAGAATTCCACT 3′; (4) LTAG forward: 5′ AGGCAGCAAGCAATGAATCC 3′; (5) LTAG reverse: 5′ATGGCAATGCTGTTTTAGAGCAA 3′; (6) LTAG FAM probe: 5′ CCACCCCAGCCATAT 3′; (7) VP3 forward: 5′ CCAGGAGGTGCAAATCAAAGA 3′; (8) VP3 reverse: 5′ CCCGTACAACCCTAAAAGTAAAGG 3′; (9) VP3 NED probe: 5′ CTGCTCCTCAATGGA 3′; (10) GAPDH forward: 5′ GAAGGTGAAGGTCGGAGTC 3′; (11) GAPDH reverse: 5′ GAAGATGGTGATGGGATTTC 3′; (12) GAPDH FAM probe: 5′ TGGAATCATATTGGAACATG 3′.

### Western blot analysis

SVG-A cells stably expressing LentiCRISPR/gRNAs were infected with JCPyV-MAD1 (5 × 10^7^ genome copies/ml) for 5 days. Equal amount of cell lysates for each condition was resolved using 12.5% SDS-PAGE and wet transferred onto a PVDF membrane (Pall). The membrane was blocked with 5% nonfat milk in 1× PBS with 0.05% Tween-20 (PBST) for 30 min and incubated with anti-VP1 PAB597 mouse monoclonal antibody and rabbit anti-cytoskeletal actin affinity purified antibody (Bethyl) in 5% nonfat milk in PBST overnight at 4 °C, followed by washes and incubation with horseradish peroxidase-labeled secondary antibody against mouse or rabbit IgG (GE Healthcare), for detection of VP1 or actin, respectively. The signal was developed using LumiGLO Chemiluminescent Substrate Kit (KPL) and imaged using the Alphaimager 2000.

### EdU assay to measure JCPyV DNA replication

HuK(i)G10 cells infected with JCPyV-MAD1 (2.6 × 10^8^ genome copies/ml) virus for 7 days were incubated with medium containing 10 μM EdU for 20 min and the newly synthesized DNA was determined by EdU incorporation using the Click-it PLUS EdU imaging kit (Molecular Probes). At least 15 fields per sample were imaged using the high content imaging platform Operetta (Perkin Elmer) at 20x magnification and images analyzed using the Columbus software (Perkin Elmer).

### JCPyV sequence analysis

The JCPyV-archetype genome was transfected into SVG-A-LentiCRISPR control cells, SVG-A- LentiCRISPR/gRNA-NCCR-a and SVG-A-LentiCRISPR/gRNA-VP1-b cells. At day 5-post transfection, viral DNA was extracted using the QlAmp MiniElute Virus Spin Kit (Qiagen), DpnI digested, and subjected to PCR and cloning using procedures described for Guide-it Indel Indentification Kit (Clontech Laboratories). The NCCR-a target regions were amplified using NCCR forward (5′CGGTACCCGGGGATCCATGACAGGAATGTTCCCCCATG3′) and reverse primers (5′CGACTCTAGAGGATCTTTTCCCGTCTACACTGTCTTCAC3′); PCR reactions for the VP1-b target region were performed using VP1-t forward (5′CGGTACCCGGGGATC CCAAATGTGCAATCTGGTGAATTT3′) and reverse primers (5′CGACTCTAGAGGATCAGTTGCTTGCCCATTAGAGTGC3′). For each sample, 50–70 colonies were picked for colony PCR using Colony PCR forward (5′ACGTTGTAAAACGACGGCCAGTGA3′) and reverse primers (5′CAATTTCACACAGGAAACAGCTATGACC3′) and the PCR products were purified using the Zymogen DNA Clean& Concentrator-5 (Zymo Research) system and sequenced using colony PCR forward primer. Valid viral sequences contained at least 100 base pair (bp) matches with the wild type JCPyV sequences at both the 5′ and 3′ ends.

## Additional Information

**How to cite this article**: Chou, Y.-y. *et al*. Inhibition of JCPyV infection mediated by targeted viral genome editing using CRISPR/Cas9. *Sci. Rep*. **6**, 36921; doi: 10.1038/srep36921 (2016).

**Publisher’s note**: Springer Nature remains neutral with regard to jurisdictional claims in published maps and institutional affiliations.

## Figures and Tables

**Table 1:** The sequences and location of gRNA targeting JCPyV genome.

Name	JCPyV genomic target	Sense or anti-sense	Target location
NCCR-a	CAAGCATGAGCTCATACCTA	Sense	NCCR TandemRepeat (50, 148)
NCCR-b	TTTACTGGCTGTTAGCTGGT	Anti-sense	NCCR TandemRepeat (78, 177)
VP1-a	AAACCCCTAAGATGCTCATC	Anti-sense	VP1 ORF (1622)
VP1-b	GAAGTTTTGGAACTTGCACG	Anti-sense	VP1 ORF (1507)
VP2	GGTGCCGCACTTGCACTTTT	Sense	VP2 ORF (529)

Numbering is according to the Mad-1 strain of JCPyV (GeneBank accession no. NC_001699).
